# Peer Review of “Patient Recommendations for the Content and Design of Electronic Returns of Genetic Test Results: Interview Study Among Patients Who Accessed Their Genetic Test Results via the Internet”

**DOI:** 10.2196/37323

**Published:** 2022-05-31

**Keywords:** user-centered design, genomic medicine, patient portals, electronic health records, return of results, bioethics, EHR, genetics, genetic testing, patient preferences, design, human factors

*This is a peer-review report submitted for the paper “Patient Recommendations for the Content and Design of Electronic Returns of Genetic Test Results: Interview Study Among Patients Who Accessed Their Genetic Test Results via the Internet."*

## Round 1 Review

### General Comments

This paper [1] describes the results of an interview study of patients regarding preferences for receiving genetic test results through an electronic patient portal. All participants had already had a genetic test and were active users of their patient portal. Some suggestions/comments to consider to improve manuscript:

### Specific Comments

#### Major Comments

##### Introduction

1. The actual purpose and study rationale/goal of the study was not described until the middle of the *Methods* section (minus the abstract). At the end of the *Introduction*, no information about the study was provided, and so, I was a little lost when transitioning from the *Introduction* to the *Methods* section for a study that hadn’t been mentioned at all. The second sentence in the *Data Collection* section could be moved up as the last sentence of the Introduction.
2. Toward the end of the *Introduction*, the inclusion about barriers to the utilization of patient portals is very broad and not specific to genetics. I would suggest limiting it to genetic test results.

##### Methods

1. Perhaps include a *Study Overview* section before *Participant Recruitment* if you do not wish to introduce the study in the *Introduction*.
2. Either provide the semistructured interview guide or provide more detail about the content and structure (eg, funnel approach?).
3. There is no mention of the analysis of content-related themes in the *Data Analysis* section.

##### Results

1. Confirm whether the patient demographics were the same for both study groups. Perhaps redo the table to include a breakdown of demographics between the two groups.
2. Clarify if the content recommendations came from the group that was asked to compare their experiences receiving genetic vs nongenetic test results through a patient portal.
3. Did you conduct any analysis to factor in patients’ background (eg, education, gender, age) or the specific type of experience with genetic testing to provide some context of their responses?
4. Without a better understanding of what the questions were, it is not totally clear if the questions were totally open-ended or if you asked them to provide feedback on specific suggestions (like the summary sheet). I assume the questions were more open-ended, given the data analysis description, but the results appear to be narrowly confined.
5. It seems to me that design recommendation #3 about smartphone functionality is not specific to genetics and should not be reported as a recommendation.
6. Some confusion about recommendations—is a simple coversheet (design recommendation #1) the same as an electronic summary (design recommendation #2) and a patient-friendly results summary (domain 2 subheading, content recommendations #2-#4).

##### Discussion

1. Include some discussion of the implementation of the recommendations. Many would take considerable time to complete for multiple testing vendors/lab reports. Are they really feasible? Do you anticipate that the laboratories will do some of this work or will it fall to test orderer?
2. In the section *Comparison to Prior Work*, I would suggest including more discussion about the format and design of current lab reports. Many are made available through labs on their websites. It is difficult to generalize lab reports for different indications/purposes and come up with a best fit with respect to design/formatting. Certainly, patient feedback will be valuable for learning how to improve the comprehension of genetic testing lab reports. Many results cannot be analyzed without the consideration of more clinical information. Test reports are intended for health providers, and thus the style, jargon, and information will understandably differ for patients. The authors should consider reviewing reports intended for patients (eg, 23andMe), which are delivered electronically.

#### Minor Comments

1. Remove the extra numbers outside at the bottom of table.
